# Native entomopathogenic *Metarhizium* spp. from Burkina Faso and their virulence against the malaria vector *Anopheles coluzzii* and non-target insects

**DOI:** 10.1186/s13071-018-2796-6

**Published:** 2018-03-27

**Authors:** Etienne Bilgo, Brian Lovett, Raymond J. St. Leger, Antoine Sanon, Roch K. Dabiré, Abdoulaye Diabaté

**Affiliations:** 1 Institut de Recherche en Sciences de la Santé/Centre Muraz, Bobo-Dioulasso, Burkina Faso; 2 Department of Entomology, University of Maryland, College Park, Maryland USA; 3 Laboratoire d’Entomologie Fondamentale et Appliqué/UFR-SVT/Université Ouaga 1, Pr. Joseph KI-Zerbo, Ouagadougou, Burkina Faso

**Keywords:** *Metarhizium*, Entomopathogenic fungi, Mosquitoes, Vector control, Honeybees, Cockroaches, Malaria, Burkina Faso

## Abstract

**Background:**

Genetically enhanced *Metarhizium pingshaense* are being developed for malaria vector control in Burkina Faso. However, not much is known about the local prevalence and pathogenicity of this fungus, so we prospected mosquitoes and plant roots (a common habitat for *Metarhizium* spp.) for entomopathogenic fungi.

**Results:**

Our investigations showed that *Metarhizium* spp. represented between 29–74% of fungi isolated from plant root rhizospheres in diverse collection sites. At low spore dosages (1 × 10^6^ conidia/ml), two mosquito-derived *M. pingshaense* isolates (Met_S26 and Met_S10) showed greater virulence against *Anopheles coluzzii* (LT_80_ of ~7 days) than isolates tested in previous studies (LT_80_ of ~10 days). In addition, the local isolates did not cause disease in non-target insects (honeybees and cockroaches).

**Conclusions:**

Our work provides promising findings for isolating local *Metarhizium* strains for application in mosquito biological control and for future transgenic biocontrol strategies in Burkina Faso.

**Electronic supplementary material:**

The online version of this article (10.1186/s13071-018-2796-6) contains supplementary material, which is available to authorized users.

## Background

Unlike mosquitocidal bacteria and viruses, ascomycete fungi can infect and kill insects without being ingested. As with chemical insecticides, tarsal contact alone is sufficient to kill mosquitoes [1]. Despite intensive efforts to develop entomopathogenic fungi as biocontrol agents against malaria vectors, the strains under investigation have not met expectations due to their poorer efficacy relative to cheaper chemical insecticides [2]. The United States Department of Agriculture (USDA) ARSEF collection (the world’s largest collection of entomopathogenic fungi) has more than 12,000 isolates of insect pathogenic fungi. Of these, only 156 are from sub-Saharan Africa (South Africa and Benin are the source of 40 and 36 isolates, respectively), with none from Burkina Faso. The mosquitocidal activity of *Metarhizium* has been enhanced by engineering them to express insect-selective neurotoxins [3–5], and a transgenic strain of *Metarhizium pingshaense* is being evaluated in semi-field trials in Burkina Faso [5]. We speculate that future development of transgenic fungi worldwide will preferentially use local isolates as these may be better adapted to kill local mosquitoes and survive harsh local conditions (i.e. rainy season heat, sunlight and humidity) than exotic strains. However, the distribution and properties of indigenous Burkinabe *Metarhizium* spp. have not been characterized. The first objective of this study was to prospect for the presence and distribution of local *Metarhizium* strains. As well as prospecting mosquitoes, we also sampled rhizosphere soils (i.e. the soil in the vicinity of plant roots that is influenced by root secretions), as some *Metarhizium* spp. are abundant in the rhizosphere and may function as symbionts promoting plant growth. The plant-beneficial effects of *Metarhizium* species correlate with their association with roots and are mediated *via* plant hormones [6]. The second objective was to evaluate the pathogenicity of local *Metarhizium* isolates against wild-caught, insecticide-resistant *Anopheles coluzzii*. Finally, we also assessed the pathogenicity of the local isolates against American cockroaches and honeybees as representative non-target or beneficial species.

## Methods

### Fungal collection, isolation and morphological identification

**Fig. 1:**
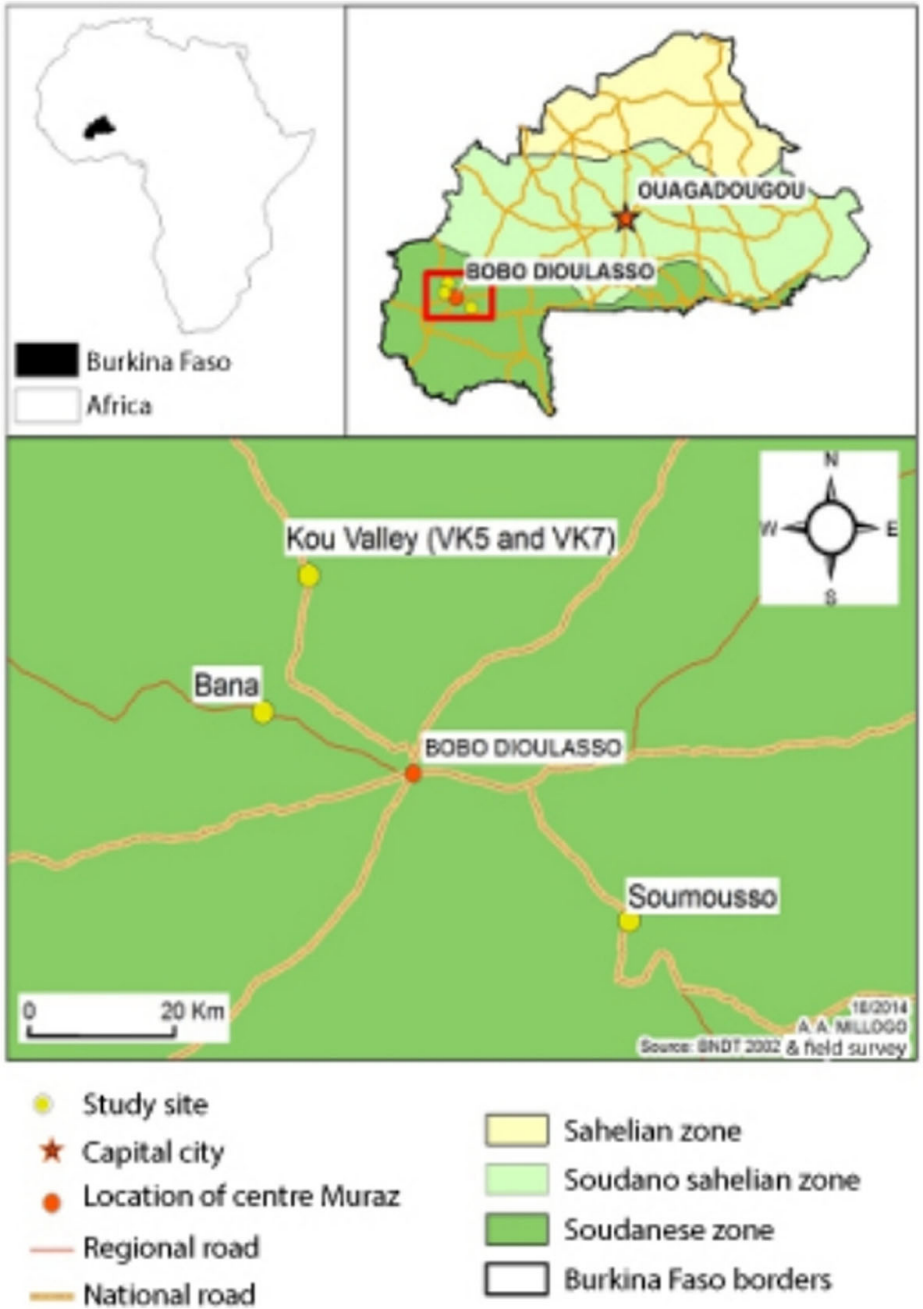
Rhizosphere and mosquito collection sites

Collections were carried out on a monthly basis during the 2015 rainy season (from July to September) from plant roots and wild-caught mosquitoes. Our three collection sites were the Kou Valley (11°23'N, 4°24'W), a rice crop area; Bana (11°9'41"N, 4°10'30"W), a savanna and forested area; and Soumousso (11°04'N, 4°03'W), a savanna and corn crop area (Fig. 1). One hundred and fifty-five plants were sampled from these three different agro-ecological sites. We followed the protocol described in [7] to collect rhizosphere soil and isolate fungi. The fungal selective medium contained 42 g potato-dextrose agar, 0.5 g chloramphenicol and 0.6 g cetyl trimethylammonium bromide per liter.

Overall, 300 mosquitoes were collected from 3 types of resting sites (inhabited houses, abandoned houses and outdoor piles of wood). Mosquitoes were brought to the IRSS/Centre Muraz insectary, where they were fed on 6% sterile glucose *ad libitum*. Approximately 22% of collected mosquitoes (67 mosquitoes) died within 2 weeks and were plated on selective medium for fungal isolations.

Fungal isolates from rhizospheres or mosquitoes were identified using macro-morphological characters, such as conidiogenesis, estimation of radial growth, spore color and mycelia texture of the isolates on PDA media according to Humber [8]. In addition, we used microscopic morphology to identify *Metarhizium* spp. spores as described by Fernandes et al. [9]. Met_S10 and Met_S26 were confirmed as *Metarhizium pingshaense* through amplification and Sanger sequencing of the intron-rich region of translation elongation factor 1-α [10].

### Fungal virulence on mosquitoes, honeybees and cockroaches

Initial screens on mosquitoes revealed two promising isolates (Met_S10 and Met_S26) isolated from mosquito cadavers from Soumousso and Bana, respectively, that readily grew on PDA and were highly virulent (Additional file 1: Table S1): these strains were therefore chosen for further characterization.

### Bioassay on mosquitoes

For bioassays, we used *An. coluzzii* adult mosquitoes reared from larval collections at the Kou Valley, Burkina Faso. Mosquitoes from this area are known to be highly resistant to multiple insecticides [5, 11]. We carried out bioassays with local *M. pingshaense* isolates Met_S10 and Met_S26. A *M. pingshaense* strain that has been used as the foundation for development of transgenic mosquito control technologies was used as a positive control; this strain was engineered to constitutively express red fluorescent protein (RFP) [5]. Expression of RFP provides a fluorescent tag for following infection processes without altering virulence. We used an atomizer protocol for infections, as described previously [12]. Three serial concentrations were used: 1 × 10^8^; 1 × 10^7^; and 1 × 10^6^ conidia/ml. We confirmed that this inoculation technique was able to deliver a repeatable inoculating dose (mean ± SE): 276 ± 16 spores per mosquito with 1 × 10^8^; 211 ± 13 spores per mosquito with 1 × 10^7^ spores/ml; and 44 ± 3 spores per mosquito with 1 × 10^6^. Mortality was counted twice daily over two weeks.

### Bioassay on non-target insects

We bioassayed Met_S10, Met_S26 and Met_RFP against a breeding line of honeybees, *Apis mellifera adansonii* (Latreille, 1804), as well as American cockroaches, *Periplaneta americana* (Linnaeus, 1758) caught in households from Soumousso. Spore doses were 1 × 10^8^, 1 × 10^7^ or 1 × 10^6^ conidia/ml, as described previously [5]. Following treatment, insects were kept in our insectarium at 25.3 ± 1 °C and 70 ± 10% relative humidity. Mortality was counted twice daily over two weeks.

## Results and discussion

*Metarhizium* spp*.* were isolated from rhizosphere soil samples across 3 sample sites: the Kou Valley, Bana and Soumousso. From the Kou Valley and Bana, we isolated 362 and 306 soil samples, respectively. *Metarhizium* spp. comprised 28.71% (*n* = 56) of the isolates from Bana and 30.72% (*n* = 94) of the total isolated fungi from the Kou Valley. We isolated 152 fungal strains from Soumousso; of these, 113 (74.34%) were *Metarhizium*, with a mean of 1.18 isolates/gram of soil (Additional file 2: Table S2). Soumousso is a savanna and corn crop area, and the higher proportion of *Metarhizium* fungi is consistent with previous studies that reported a strong association between *Metarhizium* spp*.* and soils from cultivated habitats, particularly field crops [13–15].

**Fig. 2:**
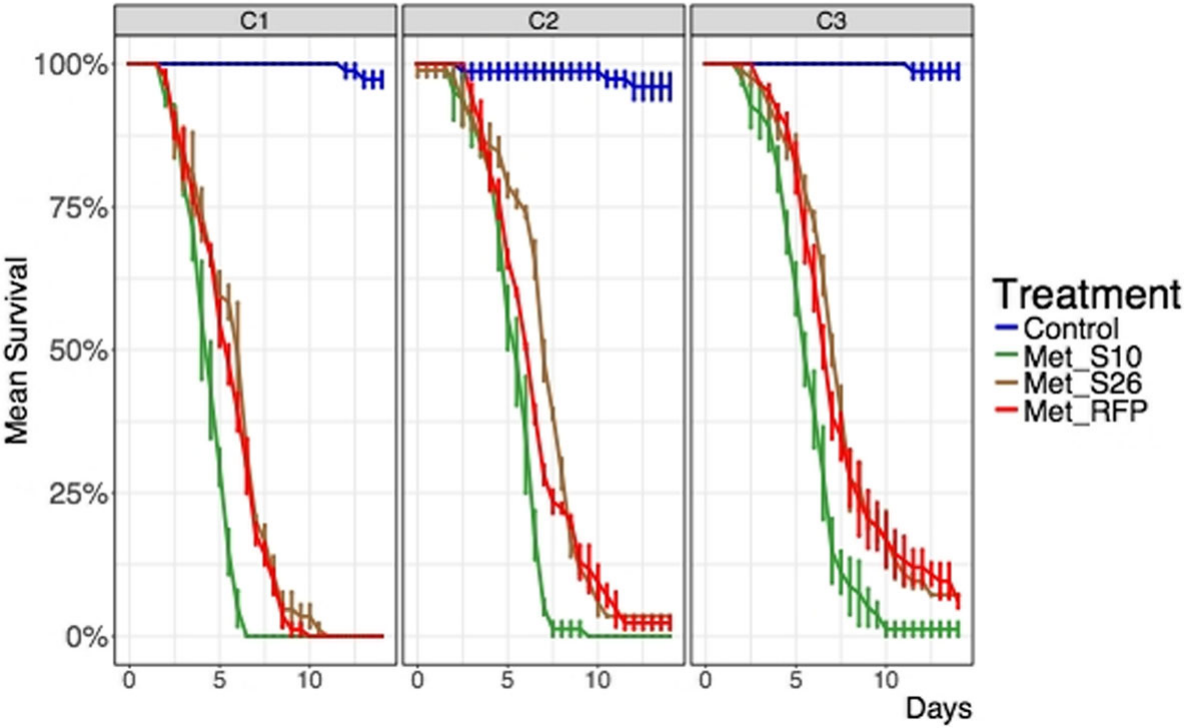
Survival curves of mosquitoes infected with Burkina Faso *Metarhizium pingshaense* isolates at different concentrations: C1, 1 × 10^8^ conidia/ml; C2, 1 × 10^7^ conidia/ml; C3, 1 × 10^6^ conidia/ml

Isolates of *Metarhizium* spp*.* represented ~1% (8/801; 3 isolates from *Culex* spp*.* and 5 isolates from *Anopheles gambiae* (*sensu lato*) of the fungi isolated from mosquitoes. Fifteen colonies of *Beauveria* spp. were isolated on mosquitoes (5 isolates from *Aedes aegypti* and 10 isolates from *Anopheles gambiae* (*s*.*l*.) at Soumousso). *Trichoderma* was the predominant genus at all sites being isolated from 56% (Vallée du Kou) to 79% (Soumousso) of mosquitoes (Additional file 2: Table S2). However, two *Metarhizium* isolates (Met_S10 and Met_S26), collected from *Anopheles gambiae* (*s*.*l*.), in an inhabited house in Soumousso and in a woodpile in Bana, respectively, were more virulent against mosquitoes than other isolates, including those from rhizospheres (Additional file 1: Table S1). At 1 × 10^8^ and 1 × 10^7^ conidia/ml, both strains achieved lower LT_50_ than Met_RFP (LT_50_ of ~6 days) [16]. At the highest concentration (1 × 10^8^ conidia/ml), the LT80 of Met_S10 (5.67 **±** 0.17 days) was significantly lower than both Met_26 (LT80 = 7.50 ± 0.29 days; Welch *t* = -5.5, *df* = 3.2, *P* = 0.01) and Met_RFP (7.17 ± 0.17 days; Welch *t* = -6.364, *df* = 4, *P* = 0.003). At the lowest concentration (1 × 10^6^ conidia/ml), Met_S10 still had a significantly (Welch *t* = -5.1962, *df* = 3.2, *P* = 0.011) lower LT80 (7.00 **±** 0.29 days) compared to Met S26 and Met_RFP, which both had LT80’s of 10 days (Fig. 2, Table 1). At intermediate concentrations, all strains achieved 80% mortality, which is the threshold value from the World Health Organization Pesticide Evaluation Scheme (WHOPES) for successful control with insecticides [17]. Thus, our results revealed higher virulence for the native isolate Met_S10, against wild-caught, insecticide-resistant *Anopheles coluzzii*. The virulence of these isolates to mosquitoes is also higher than isolates from Benin and in Kenya where *Metarhizium anisoplae* strains were originally isolated from a white fly, *Trialeurodes vaporariorum* [16, 18].

**Table 1:** LT_80_s and grouping LT_80_ values for *Anopheles coluzzii* adults treated with Burkina Faso local *Metarhizium pingshaense* strains (Met_10 and Met_26) compared with wild type *Metarhizium pingshaense* expressing red fluorescent protein (Met_RFP) at three different concentrations

Concentration (conidia/ml)^a^	Treatment	LT_80_ + SE (days)	Grouping LT_80_^b^
C1 (1 × 10^8^)	Met_S10	5.67 ± 0.167	a
	Met_S26	7.50 ± 0.289	b
	Met_RFP	7.18 ± 0.167	b
C2 (1 × 10^7^)	Met_S10	6.67 ± 0.167	a
	Met_S26	8.67 ± 0.167	b
	Met_RFP	8.83 ± 0.167	b
C3 (1 × 10^6^)	Met_S10	7.00 ± 0.289	a
	Met_S26	10.00 ± 0.500	b
	Met_RFP	10.00 ± 1.041	b

*Abbreviation*: *SE* standard error of the mean

^a^In 0.01% Tween80

^b^Pairwise comparison of LT_80_ values per spraying conidia suspension concentrations; treatments with no letters in common differ significantly at *P* < 0.05

We bioassayed honeybees and cockroaches with the local strains and Met_RFP. However, even at the highest spore dosage (1 × 10^8^ conidia/ml), these fungi did not significantly increase mortality compared to controls containing no conidia (Table 2). Fewer than 5% of honeybees and cockroaches died during the bioassays, and no mycosis was observed on any cadavers. This is in agreement with previous studies that report Met_RFP is a specialist to Culicidae [5]. The host ranges of different *Metarhizium* strains are chiefly controlled by recognition events on the cuticle [19], and the cuticles of honeybees, cockroaches and mosquitoes would likely have many topographical and chemical differences.

**Table 2:** Two week-survival and grouping survival values for *non-target insects* (Honeybees and Cockroachs) treated with Burkina Faso local *Metarhizium pingshaense* strains (Met_10 and Met_26) compared with wild type *Metarhizium pingshaense* expressing red fluorescent protein (Met_RFP) at three different concentrations and a control (0.01% Tween)

Non-target insect	Concentration (conidia/ml)^a^	Treatment	Survival + SE (%)	Grouping survival^b^
Honeybee	C1 (1 × 10^8^)	Control	93.8 ± 1	a
		Met_RFP	98.2 ± 1	a
		Met_S10	98.1 ± 2	a
		Met_S26	94.6 ± 2	a
	C2 (1 × 10^7^)	Control	94.6 ± 1	a
		Met_RFP	97.3 ± 2	a
		Met_S10	98.3 ± 1	a
		Met_S26	97.3 ± 0	a
	C3 (1 × 10^6^)	Control	95.3 ± 1	a
		Met_RFP	99.1 ± 1	a
		Met_S10	99.0 ± 0	a
		Met_S26	95.1 ± 2	a
Cockroach	C1 (1 × 10^8^)	Control	95.7 ± 2	a
		Met_RFP	97.8 ± 2	a
		Met_S10	98.8 ± 1	a
		Met_S26	97.5 ± 1	a
	C2 (1 × 10^7^)	Control	96.1 ± 2	a
		Met_RFP	97.5 ± 1	a
		Met_S10	98.7 ± 1	a
		Met_S26	97.7 ± 1	a
	C3 (1 × 10^6^)	Control	96.0 ± 1	a
		Met_RFP	97.0 ± 1	a
		Met_S10	97.0 ± 1	a
		Met_S26	96.0 ± 1	a

*Abbreviation*: *SE* standard error of the mean

^a^In 0.01% Tween80

^b^Pairwise comparison of survival mean values per spraying conidia suspension concentrations; treatments with no letters in common differ significantly at *P* < 0.05

Despite being more virulent than other WT *Metarhizium* strains, the Burkinabe *Anopheles*-derived isolates are still significantly less effective than transgenic strains expressing arthropod toxins [5]. However, our results suggest that these native Burkinabe *Metarhizium* strains would make attractive candidates for transgenic virulence enhancement and subsequent use as transgenic biocontrol agents.

## Conclusion

Native fungal isolates may offer a superior alternative to introducing a foreign biocontrol strain, as they may be better adapted to both kill local mosquitoes and survive local conditions. There are also regulatory and ecological advantages to using strains already present in the country or in the ecosystem. This study provides a promising precedent for isolating local *Metarhizium* strains for application in mosquito biological control, and it lays a foundation for future transgenic biocontrol projects in Burkina Faso.

## Additional files

Additional file 1: **Table S1.** Preliminary infections data on mosquitoes. (XLSX 34 kb)

Additional file 2: **Table S2.** List of fungal strains isolated from rhizosphere and mosquitoes. (XLSX 72 kb)

## Abbreviations

Met_S10: *Metarhizium pingshaense* strain No. 10
Met_S26: *Metarhizium pingshaense* strain No. 26
Met_RFP: *Metarhizium pingshaense* expressing red fluorescent protein (RFP)
LT_50_: Median (50%) lethal time after exposure to fungal infections
LT_80_: 80% lethal time after exposure to fungal infections
SE: Standard error
